# Effects of Nursing Quality Improvement on Thrombolytic Therapy for Acute Ischemic Stroke

**DOI:** 10.3389/fneur.2018.01025

**Published:** 2018-11-29

**Authors:** Zhuo Liu, Yingkai Zhao, Dandan Liu, Zhen-Ni Guo, Hang Jin, Xin Sun, Yi Yang, Huijie Sun, Xiuli Yan

**Affiliations:** ^1^Cadre Ward, The First Hospital of Jilin University, Changchun, China; ^2^Physical Examination Center, The First Hospital of Jilin University, Changchun, China; ^3^Department of Neurology, The First Hospital of Jilin University, Changchun, China

**Keywords:** stroke nurse, nursing quality improvement, thrombolysis, door-to-needle time, onset-to-needle time

## Abstract

**Background and purpose:** Intravenous thrombolytic therapy significantly improves the outcomes of acute ischemic stroke patients in a time-dependent manner. The aim of this study was to investigate whether continuous nursing quality improvement in stroke nurses has a positive effect on reducing the time to thrombolysis in acute ischemic stroke.

**Methods:** The implementation of nursing quality improvement measures includes establishing full-time stroke nurses, pre-notification by emergency medical services (EMS), stroke team notification protocols, rapid triage, publicity and education, etc. Using a history-controlled approach, we analyzed acute ischemic stroke patients with intravenous thrombolysis during a pre-intervention period (April 1, 2015-July 31, 2016), trial period (August 1, 2016-October 31, 2016), and post-intervention period (November 1, 2016-September 30, 2017). This was done in accordance with the implementation of nursing quality improvement measures, including the general characteristics of the three groups, the time of each step in the process of thrombolysis, and the prognosis.

**Results:** After the implementation of nursing quality improvement measures, the median door-to-needle time (DNT) was shortened from 73 min (interquartile range [IQR] 62–92 min) to 49 min (IQR 40-54 min; *p* < 0.001) in the post-intervention period. The median onset-to-needle time (ONT) was reduced from 193 min (IQR 155–240 min) to 167 min (IQR 125-227 min; *p* < 0.001). The proportion of patients with DNT ≤ 60 min increased from 23.94% (51/213) to 86.36% (190/220; *p* < 0.001) while the proportion of patients with DNT ≤ 40 min increased from 3.29% (7/213) to 25.00% (55/220; *p* < 0.001). The median time for door-to-laboratory results was decreased from 68 min to 56 min (*p* < 0.001). There was no significant difference in the fatality rate, 90-day modified Rankin score, length of stay or hospitalization expenses between the three groups of patients (*p*> 0.05).

**Conclusions:** Implementation of nursing quality improvement measures in stroke nurses is an important factor in shortening the time of medication in patients with thrombolytic therapy, reducing the delay of intravenous thrombolysis in the hospital and helping to expedite presenting patients' arrival to the hospital post-stroke.

## Introduction

Stroke has become the most frequent cause of death in China (1). The latest Ness-China (National Epidemiological Survey of the Stroke in China) study revealed that (2) the incidence of stroke in China is estimated at 274.4/100,000 (3), of which acute ischemic stroke (AIS) accounted for 60–80% of cases. The most effective treatment currently is intravenous thrombolytic therapy with recombinant tissue-type plasminogen activator (rt-PA). However, a large number of randomized controlled trials have shown that the therapeutic efficacy of rt-PA is strictly time-dependent (4–7); for every 15 min of time to thrombolytic therapy that is saved, on average, there is an additional 1 month of healthy life (8). Therefore, it is essential to provide assessment and treatment for AIS patients as soon as possible.

The International SITS WATCH study aims for a median door-to-needle time (DNT) of 40 min (9). However, two studies in China have shown that the DNT in most hospitals significantly exceeded the maximum standard (10, 11) and was even twice the time in many European countries (12). Previous studies have reported the effectiveness of several strategies for reducing DNT times in acute ischemic stroke patients, including rapid triage, stroke team notification protocol, rapid laboratory testing, etc. (13, 14). In recent years, some hospitals in China have also begun to adopt the re-engineering process to shorten in-hospital treatment delays and have obtained some positive but preliminary progress (15, 16). To assure that more patients have timely thrombolytic therapy, the stroke center at the First Hospital of Jilin University implemented a series of measures to shorten the DNT, such as establishing full-time stroke nurses, pre-notification by emergency medical services (EMS), and stroke team notification protocol, rapid triage, rapid acquisition, and interpretation of brain imaging, etc. We found that the implementation of these nursing quality improvement measures resulted in substantial and positive effects on shortening the treatment process after AIS patients arrived at our hospital.

## Methods

The study design was approved by the ethics committee of the First Hospital of Jilin University. All participants gave written informed consent.

### Design and participants

**Figure 1:**
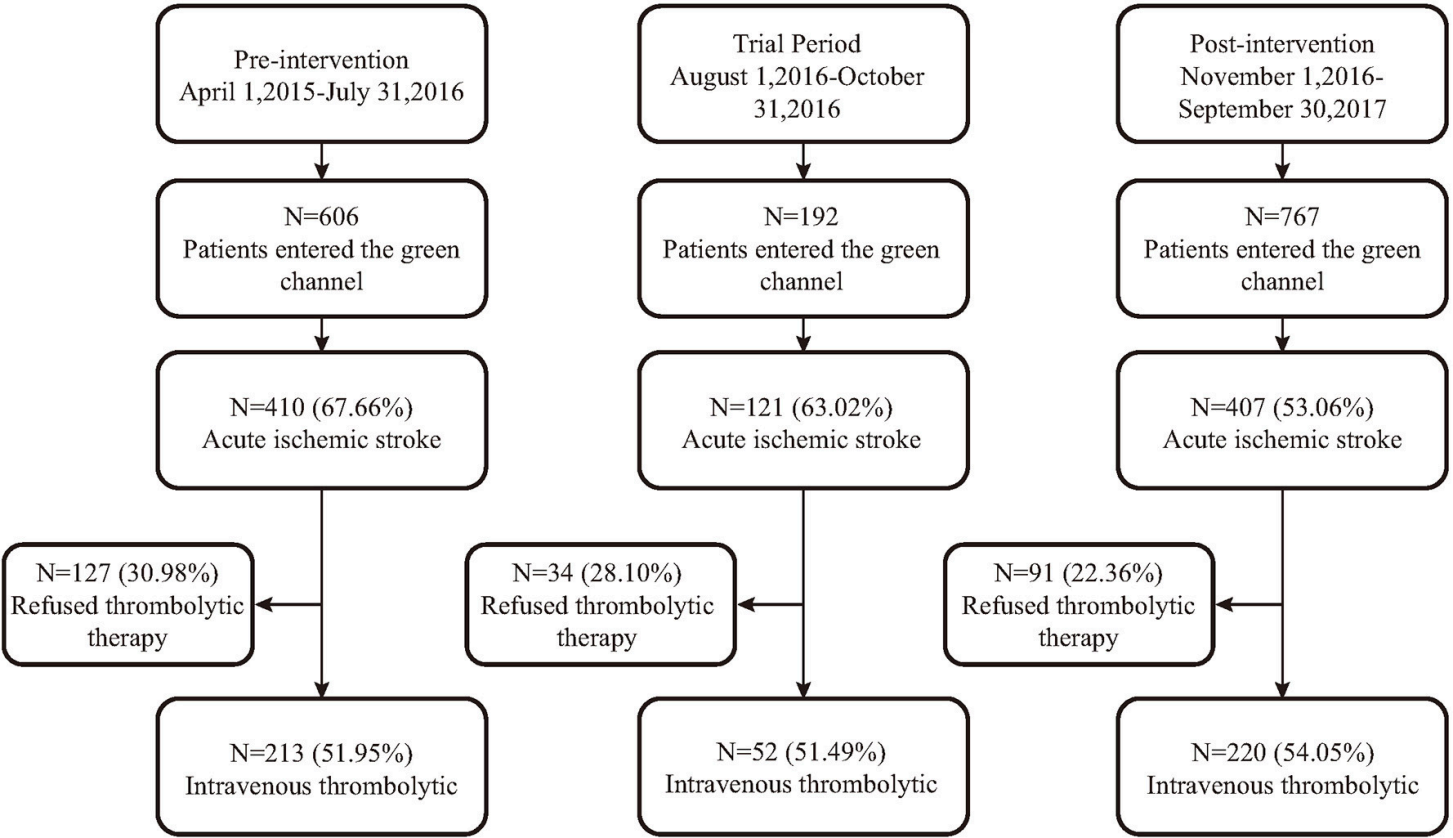
Summary of number of patients who entered the green channel in the three time periods.

The Department of Neurology in the First Hospital of Jilin University established a follow-up central electronic database for patients with AIS. It records the total number of patients treated with thrombolysis and the entire diagnosis and treatment process, including the relevant indicators of diagnosis, and treatment and the time spent in each medical process, among other factors. In this study, a historical control analysis was adopted to select the patients with AIS who were hospitalized with rt-PA treatment in the emergency green channel (a special fast pass for thrombolytic therapy) from April 1, 2015 to September 30, 2017 at our hospital. The patients were set in groups along the following lines: the pre-intervention period (April 1, 2015-July 31, 2016), trial period (August 1, 2016-October 31, 2016) and the post-intervention period (November 1, 2016-September 30, 2017) (Figure 1). All of the patients met the following criteria: both genders and age ≥18 years; conformed to the diagnostic criteria of Guidelines for the Early Management of Patients with Acute Ischemic Stroke (17); stroke onset time < 4.5 h and accepted computed tomography (CT) diagnosis of the brain; completed a telephone follow-up survey 3 months after discharge; and the patient or family member signed an informed consent letter.

### Nursing quality improvement measures

#### Establish a full-time stroke nurse

**Figure 2:**
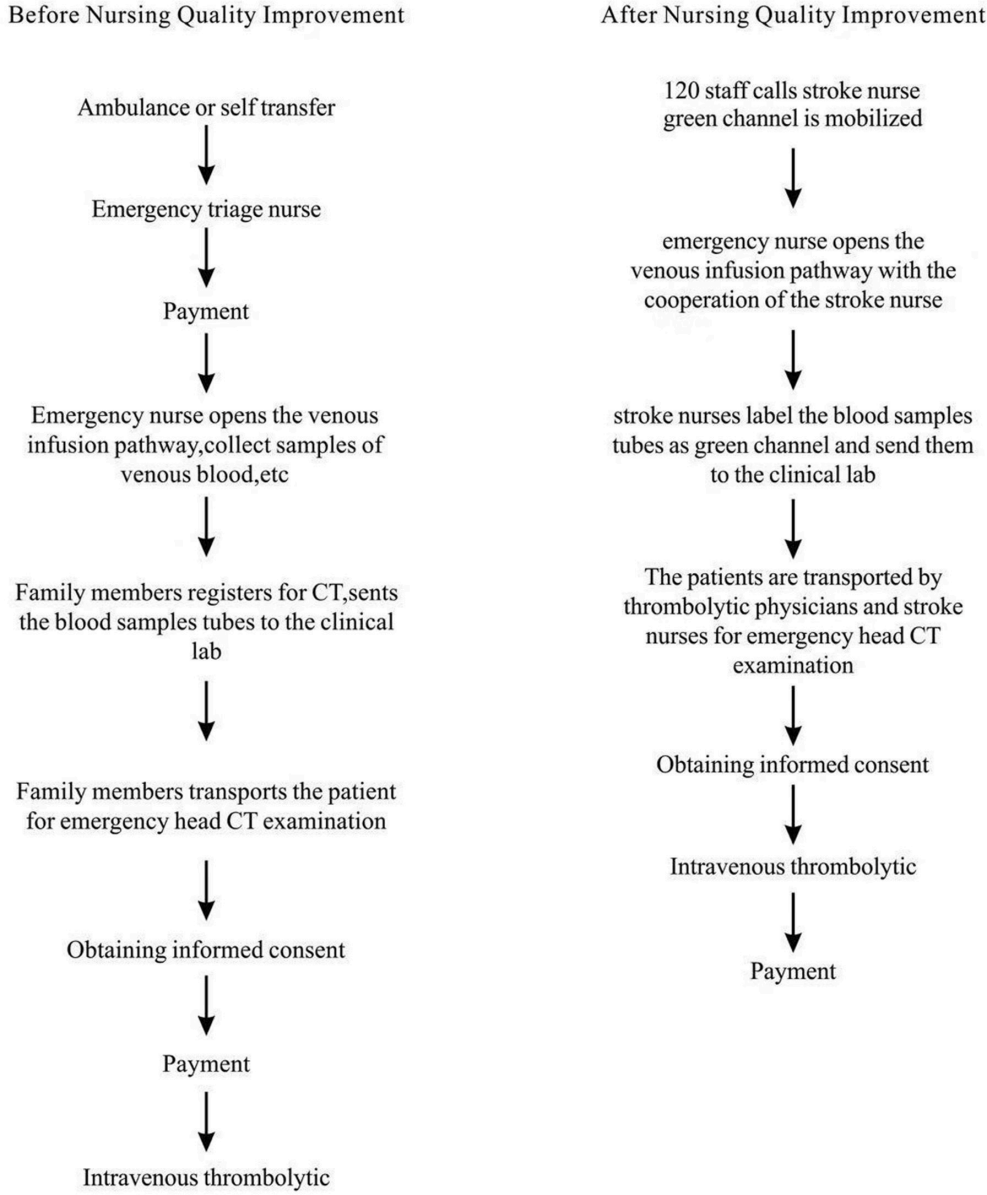
The work flow of the green channel before and after nursing quality improvement.

The position of a stroke nurse is more mature in developed countries in Europe and has clear job responsibilities (18). According to our center's requirements, three stroke nurses were trained for three months prior to induction. They were in charge of the entire caring process from the patient's emergency admission to the end of intravenous thrombolysis (Figure 2).

#### Pre-hospital and stroke team notification protocol

Our center established a close cooperative relationship with the Changchun City Emergency Center in January 2017 and set up an online instant messenger group for the First Hospital of Jilin University-emergency thrombolytic linkage. Stroke nurses in the group release daily thrombolytic information, review and respond in a timely manner to the first aid personnel's information on suspected stroke patients, and activate thrombolytic physicians and nurses at the time of pre-notification. Once a stroke is confirmed, the emergency green channel in the hospital is mobilized to receive the patient. The instant communication system also instructs first-aid personnel to open the patient's venous access, detect blood glucose at the fingertips, measure vital signs, collect medical history in ambulances, and communicate with the emergency medical service technician in real time.

#### Strengthen the quality control of nurses in multiple aspects

##### Rapid triage

Once patients arrive at the hospital, a large digital timer was adhered to the bed to show the team how much time was passing. The emergency triage nurses had the responsibility to clearly assess their condition in a very short time and provide an accurate judgment. They were trained to become familiar with the principle of FAST for the early recognition of stroke. The goal of this training was to ensure that emergency triage nurses could reach 100% recognition of stroke after examination and to shorten the initial screening diagnosis time.

##### Increase the quality control of emergency nurses

After patients enter the green channel, the emergency nurse opens the venous infusion pathway as soon as possible with the cooperation of the stroke nurse, collects samples of venous blood for hematologic tests (including blood routine, coagulation routine, blood biochemistry, troponin, and fingertip blood sugar), and conducts a bedside electrocardiograph examination. The whole process needs to be completed within 5 min. Then the stroke nurses label the blood samples tubes as green channel and send them to the clinical lab as soon as possible.

##### Rapid acquisition and interpretation of brain imaging

The patients are transported by thrombolytic physicians and stroke nurses for emergency head CT examination in coordination with the radiology department for priority opening of green channels. The goal is to limit the waiting time to a CT scan for stroke patients to < 2 min.

#### Strengthening publicity and education

The recognition rate of early symptoms of stroke and the awareness of thrombolytic therapy in general in Chinese residents are low (19, 20). Studies have shown that improving the public's awareness of stroke symptoms can prompt individuals to activate EMS in critical situations, which reduces pre-hospital delays (21).

##### Disseminating stroke information

The nursing group at our center compiled a variety of brochures, including basic knowledge of stroke prevention, inducing factors, risk factors, knowledge of emergency treatment after onset, and knowledge of thrombolytic treatment. These brochures were provided free of charge during each educational activity and to patients and their families at the reading corner in the treatment area. We established an online WeChat public group, Cerebrovascular Disease Center of the First Hospital of Jilin University, and the nursing group was responsible for publishing a post on the popularization of stroke science written by the nursing staff every 2 weeks.

##### Health education in the treatment area

The nurses give special lessons on various stroke subjects in the treatment area's classroom using multimedia equipment at a fixed time in the afternoons. The contents include drug therapy, self-monitoring, healthy diet, and early exercise rehabilitation therapy. We also organize a weekly departmental mission to increase the knowledge of patients and their families on the prevention and treatment of stroke.

##### Free community consultation

The nursing staff organizes “Red Hand Ring” volunteer activities in every major community in our city each month, conducts health talks and counseling, distributes free brochures, and provides a free assessment of blood pressure, blood lipids, blood sugar, and other parameters to improve residents' understanding of a healthy life and disease prevention knowledge.

### Outcome measures

The main outcome of this study is the median DNT during the three study periods described previously. Other secondary outcome indicators include the proportion of DNT ≤ 60 min, DNT ≤ 40 min, onset to needle time (ONT), door-to-CT time, door-to-laboratory results time, the proportion of the 90-day modified Rankin score (mRS) ≤ 2, fatality rate, length of stay and hospitalization expenses of patients.

### Statistical analysis

The statistical analyses were carried out using IBM SPSS statistics version 22.0. Descriptive analysis of the general data and the measurement data in accordance with the normal distribution is expressed as the mean ± standard deviation (x ± s), and the comparison between the three groups used the SNK-q test. The measurement data and grade data that do not conform to a normal distribution are described by median and quartile range, whereas the comparison for the three groups used the Kruskal-Wallis test. The counting data are expressed as a ratio of rate and composition, analyzed with χ^2^ tests. A 2-tailed *p* < 0.05 indicated statistical significance.

## Results

### Baseline characteristics

In total, 485 acute ischemic stroke patients (213 in the pre-intervention period, 52 in the trial period, and 220 in the post-intervention period) were enrolled in this study. More patients entered the green channel for thrombolytic therapy after the implementation of the new nursing strategies (Figure 1). Table 1 compares the baseline characteristics of the patients who were treated before the implementation of the nursing quality improvement measures to those treated afterward. The mean patient age was 60.5 ± 11.9 years, and 350 (72.16%) of the patients were men.

**Table 1:** Baseline characteristics.

**Characteristics**	**Pre-intervention (*n* = 213)**	**Trial period (*n* = 52)**	**Post-intervention (*n* = 220)**	**Statistic**	***P***
Age(y),mean±SD	60.71 ± 12.63	60.06 ± 9.89	60.47 ± 11.61	0.069	0.933^•^
Male(%)	72.77	76.92	70.45	0.945	0.623^▴^
Smoking(%)	64.32	69.23	56.36	4.453	0.108^▴^
Drinking(%)	48.36	50.00	45.45	0.547	0.761^▴^
Hypertension(%)	49.30	36.54	21.36^*^	37.032	< 0.001^▴^
*NIHSS,(median IQR)*	10 (6-15)	10 (5-14)	8 (4-12)^*^	20.224	< 0.001^■^
**PAST MEDICATIONS**					
Antiplatelet(%)	23.47	11.54	4.10^*^	35.208	< 0.001^▴^
Anticoagulants(%)	2.35	0	0.45	2.870	0.209^▴^
**LABORATORY DATA**					
Glucose(mg/dl)	5.85 (5.17-7.11)	5.51 (4.88-6.64)	5.89 (5.08-7.55)	2.112	0.348^■^
Platelet( × 103/μl)	207 (177-239)	203 (178-260)	199 (156-240)	3.996	0.136^■^

* *P < 0.01 vs. pre-intervention*.

• *Groups were compared by SNK-q test*.

■ *Groups were compared by Kruskal-Wallis test*.

▴ *Groups were compared by χ^2^ test*.

### Primary and secondary outcome analysis

#### Pre-intervention period vs. post-intervention period

Compared to the pre-intervention period, DNT and ONT both significantly decreased in the post-intervention period: 73 min vs. 49 min (*p* < 0.001) and 193 min vs. 167 min (*p* < 0.001), respectively. The percentage of DNT ≤ 60 min increased from 23.94 to 86.36% (*p* < 0.001) and DNT ≤ 40 min was increased from 3.29 to 25.00% (*p* < 0.001) after the nursing quality improvements.

Of note, the median door-to-laboratory results time decreased from 68 min (56–79 min) to 56 min (47–69 min) in the post-intervention period (*p* < 0.001). There was no significant difference in the door-to-CT time (*p* = 2.806) before and after the improvement in nursing quality.

There were no differences in fatality rate, 90-day mRS, length of stay or hospitalization expenses between the two groups. Table 2.

**Table 2:** Comparison of important treatment parameters, M (P_25_, P_75_).

**Outcome**	**Pre-intervention (*n* = 213)**	**Trial period (*n* = 52)**	**Post-intervention (*n* = 220)**	**statistic**	***P***
Door-to-needle time (min)	73 (62–92)	70 (60–86)	49 (40–54)^*^^#^	200.493	< 0.001^■^
DNT ≤ 60 min (%)	23.94	32.69	86.36^*^^#^	179.190	< 0.001^▴^
DNT ≤ 40 min (%)	3.29	1.92	25.00^*^^#^	51.456	< 0.001^▴^
Door-to-CT time (min)	25 (18–33)	26 (20–35)	23 (17**-**33)	2.806	0.246^■^
Door-to-laboratory	68 (56**-**79)	65 (51–78)	56 (47–69)^*^^#^	34.501	< 0.001^■^
results time (min)				
Onset-to-needle time (min)	193 (155–240)	190 (150–234)	167 (125–225)^*^	16.618	< 0.001^■^
90-day mRS ≤ 2(%)	62.91	65.38	65.00	0.246	0.884^▴^
Fatality rate (%)	7.98	5.77	5.45	4.624	0.099^▴^
Length of stay (day)	11 (8–14)	10 (9–13)	11 (8–13)	1.454	0.483^■^
Hospitalization expenses (yuan)	23554.81	22131.67	25084.50	2.363	0.307^■^

* p < 0.001 vs. pre-intervention;

# *p < 0.05 vs. trial period*.

■ *Groups were compared by Kruskal-Wallis test*.

▴ *Groups were compared by χ^2^ test*.

#### Pre-intervention period vs. trial period

There were no differences in DNT, the percentage of patients treated ≤ 40 min, ONT, the median door-to-laboratory results, fatality rate, 90-day mRS, length of stay, or hospitalization expenses between the pre-intervention period and the trial period.

#### Trial period vs. post-intervention period

Compared to the trial period, DNT and ONT both significantly decreased in the post-intervention period: 70 vs. 49 min (*p* < 0.001) and 190 vs. 167 min (*p* < 0.001), respectively. The percentage of DNT ≤ 60 min increased from 32.69% to 86.36% (*p* < 0.001) and DNT ≤ 40 min was increased from 1.92% to 25.00% (*p* < 0.001). The median door-to-laboratory results time decreased from 65 min (51–78 min) to 56 min (47–69 min) in the post-intervention period (*p* = 0.011).

There were no differences in fatality rate, 90-day mRS, length of stay, and hospitalization expenses between the two groups.

## Discussion

Our research showed that after the implementation of nursing quality improvement measures, including the establishment of fulltime stroke nurses, pre-notification by EMS, and stroke team notification protocal, rapid triage and laboratory testing, publicity and education, etc., the median DNT, ONT and door-to-laboratory result times of venous thrombolysis in patients with acute ischemic stroke significantly shortened, especially after the trial period. Our nursing quality improvement measures avoided several steps that might delay thrombolytic therapy. Finally, 86.36% of AIS patients received thrombolytic therapy within 1 h of admission after all the nursing quality improvement measures were implemented.

The overall advantage of this nursing quality improvement project lies in the integration of multiple measures to reduce pre-hospital and in-hospital delays. First, the hospital closely collaborates with EMS to establish a highly effective integrated management and treatment model. In this model, the nurses are responsible for liaising and coordinating the work, determining whether stroke patients need to be evaluated by emergency pre-screening nurses, and directing a seamless transition between pre-hospital first aid and the in-hospital green channel for thrombolytic therapy. Our working model propels the first aid platform forward and is consistent with stroke alert teams established by Jeon et al. (22) and the results of the implementation of the FAST system in the United States (23). In recent years, more patients have had access to green channels for AIS and have received prompt treatment. The second important measure is to implement health education activities both in and out of the hospital and to provide full support to the role of nurses as educators. Studies have shown that health promotion strategies can expedite hospital attendance after stroke (24). Raising the awareness of patients and their families about thrombolytic therapy can reduce the number of patients who do not receive thrombolysis because of their refusal to provide informed consent. As can be seen from our current data, the proportion of patients who did not undergo thrombolytic therapy due to rejection by family members was significantly reduced from 30.98% (127/410) to 22.36% (91/407) in the post-intervention period (*p* = 0.005; Figure 1).

In this study, the three fulltime stroke nurses were all male and had ample experience and knowledge in neurology after 3 months of specialist training. Stroke nurses have been involved in two recent revascularization projects (25, 26) for AIS patients in China. As the central part of the entire medical team, they have high levels of communication and coordination skills, coordinate various departments to improve work efficiency, and have achieved positive results. The training and development of stroke nurses in the future can constantly enrich the instruction and investigation model and pay more attention to scientific research skills, promote the development of specialist nurses, and advocate for the process of nursing specialization.

We acknowledge that our research has some limitations. First, the data were from a single center and historical controls were used for the comparison cohort. Second, in order to avoid medical disputes, we must ask the patient or family for permission and to sign informed consent forms before rt-PA administration. Thus, several methods cannot be implemented in this situation, such as delivering rt-PA in a CT scanner, pre-mixing rt-PA ahead of time, etc.

## Conclusion

The implementation of nursing quality improvement measures led by stroke nurses significantly increased the total number and proportion of AIS patients receiving thrombolytic therapy and shortened the door-to-needle time and onset-to-needle time. We showed that these measures are successful in improving the treatment of AIS patients and should be adopted by other hospitals. Further studies should explore the repeatability of protocols in other hospitals and evaluate their impact on patients' functional outcomes.

## Ethics statement

This study was carried out in accordance with the recommendations of the ethics committee of the First Hospital of Jilin University with written informed consent from all subjects. All subjects gave written informed consent in accordance with the Declaration of Helsinki. The protocol was approved by the ethics committee of the First Hospital of Jilin University.

## Author contributions

HS, XY, and YY conceived and oversaw study. DL, Z-NG, HJ, and XS performed data collection. ZL and XY performed statistical analysis. ZL and YZ wrote manuscript.

### Conflict of interest statement

The authors declare that the research was conducted in the absence of any commercial or financial relationships that could be construed as a potential conflict of interest.
